# Time to See Quality Measurement Differently: Focus on Reflection, Learning and Improvement; A Response to the Recent Commentaries

**DOI:** 10.34172/ijhpm.2022.7228

**Published:** 2022-03-15

**Authors:** Marieke Zegers, Gijs Hesselink, Hans ( J.G.) van der Hoeven

**Affiliations:** Radboud University Medical Center, Radboud Institute for Health Sciences, Department of Intensive Care, Nijmegen, The Netherlands.

 We have read the comments on our article ‘Perceived burden due to registrations for quality monitoring and improvement in hospitals: a mixed methods study.’^1^ We are pleased to read that the authors unanimously endorse our findings, and we support their suggestions (at the macro, meso and micro level) for optimizing quality measurement in healthcare and reducing administrative burden.

 We would like to take the opportunity to react on their commentary and add an important overarching focus to our topic of interest, that is: the need to refocus on quality measurement by seeing it primarily as a means to allow clinicians to reflect, learn and improve at the micro/meso level rather than as a tool for benchmarking and accountability purposes at the macro level.

 Jeurissen and colleagues rightly point out that the administrative burden and subsequent costs transcends the individual clinician level. In their words “registries also form part of a broader data ecosystem where many interdependencies and connections do apply.” Health systems in many high-income countries such as the Netherlands consist of a large and growing number of players (ie, oversight agencies, purchasers, patient organisations, accreditation organizations and data companies) each with their own responsibility and interest to collect and analyse performance data, and all working within a scattered and uncoordinated registry landscape.^2^ This fragmentation leads to a growing waste of resources.

 A holistic approach with adequate governance of data-issues and information system and consensus about a core set of measures are key to align information demands from several parties.However, we believe that adequate governance should start with the use of performance data to improve clinical practice at the local level. Excessive quality measurements for judging and certifying performance levels with limited impact on the things patients and payers want and need (better outcomes and lower costs) should be replaced by a lean and effective set of quality measurements. One that enables organisations and clinicians to continuously reflect, learn and improve their clinical practice. This can only work if clinicians feel the necessity to perform registrations (ie, because it contributes to better care in practice) and are empowered and trusted by their supervisors/boards in providing high-quality care without the need to fulfil numerous quality registrations daily whose value is often questioned. Our study showed that only 36% of all measures are useful for improving care in everyday practice, undermining direct patient care and the morale of health care providers.^3^ Therefore, the notion of quality measurement should by altered and primarily seen as a mean to allow clinicians to reflect, learn and improve in practice rather than as a tool for benchmarking and accountability purposes at the macro level.

 The tripartite framework proposed by Hysong and colleagues could be helpful in reconsidering existing measures and developing new ones based on this notion.^4^ Moreover, as Hysong and colleagues also mentioned, it encourages administrators to involve those directly involved in the delivery and receipt of care into the decision-making process for selecting appropriate measures. We hypothesize that the use of a core set of appropriate measures developed by those primarily involved and critically evaluated as proposed by Hysong and colleagues could lead to less administrative burden, more effective governance and more proactive clinicians in quality improvement.

 A refocus on the essence of quality measurement also means prioritising measures that add value to the lives of patients (eg, functional status and quality of life) and are useful for clinicians to improve their daily care (eg, needs and preferences of patients for clinical decision making), and abandoning those with low value; those that do not effectively contribute to better care, health outcomes and patient experiences. This is essential to turn the idle of health care professionals after two years of hardworking during the coronavirus disease 2019 (COVID-19) pandemic. The pandemic together with the ever-growing number of rules and performance measures has taken its toll on the morale and motivation of clinicians. The pandemic showed that these aspects are essential for the delivery of high-quality care.

 Right now, appreciation, reduction of regulatory pressure and trust are important for the intrinsic motivation of health care professionals. Disconnection should be prevented between quality measurements and those who want to use it at macro level and those who should administrate these and want to see the benefits of these registrations. We believe that professional organisations together with patient organisations have an important role in canalizing information demands for their members (eg, physicians, nurses, paramedics, and patients) and gaining trust by transparency. Transparency about the learning and improving capabilities of organisations and individual clinicians, how clinicians learn and improve and prevent risks in daily practice, should give all stakeholders (ie, patients, society, and oversight agencies) trust that clinicians are willing to provide the best care every day.

## Ethical issues

 Not applicable.

## Competing interests

 Authors declare that they have no competing interests.

## Authors’ contributions

 MZ, GH, and HH wrote and approved the final manuscript.
